# Role of Adipose Tissue Derived Exosomes in Metabolic Disease

**DOI:** 10.3389/fendo.2022.873865

**Published:** 2022-05-04

**Authors:** RuiYan Mei, WeiWei Qin, YanHua Zheng, Zhuo Wan, Li Liu

**Affiliations:** Department of Hematology, Tangdu Hospital, Fourth Military Medical University, Xi’an, China

**Keywords:** adipose tissue, exosome, obesity, diabetes, adipose inflammation

## Abstract

Adipose tissues perform physiological functions such as energy storage and endocrine, whose dysfunction will lead to severe metabolic disorders. Accumulating evidences show that exosomes can meditate communications between different tissues by transporting nucleic acids, proteins and other biological factors. More importantly, exosomes secreted by adipose tissue function as critical contributing factors that elucidate specific mechanisms in metabolic disturbance such as obesity, adipose inflammation and diabetes etc. Adipose tissue is the major source of circulating exosomal miRNAs. miRNA secreted from adipose tissues not only altered in patients with metabolic disease, but also result in an increase in metabolic organ talk. Here we have reviewed the latest progress on the role of adipose tissue derived exosomes roles in metabolic disorders. Moreover, the current obstacles hindering exosome-based therapeutic strategies have also been discussed.

## Introduction

Metabolic dysfunctions include hyperglycemia, obesity, dyslipidemia, insulin resistance, etc. (1). Adipose tissue dysfunction underlies the pathogenesis of metabolic diseases (2). Adipose tissue is not only an energy storage organ, but also an endocrine organ that regulates the metabolic homeostasis of tissues and organs throughout the body. Nearly 100 adipokines have been discovered (3). Under physiological conditions, adipokines can act on the brain, liver, skeletal muscle, cardiovascular and immune systems, and endocrine pancreas and other tissues and organs (4). When the body is in a state of metabolic disorder, the overproduction of pro-inflammatory adipokines and the decreased expression of anti-inflammatory adipokines lead to increased adipose tissue volume, adipocyte damage, degeneration, and proliferation, aggravating adipose inflammation and insulin resistance (5, 6). In addition to adipokines dysregulation, adipose tissue-derived exosomes have been found to play important roles in metabolic disorders. Early evidence suggests that adipose tissue-derived exosomes contribute to the development of metabolic disorders such as obesity and insulin resistance by regulating distant organ tissues, such as the liver and pancreas (7–9). In studying adipose tissue-derived exosomes, the researchers found that exosomes from obese adipose tissue, when applied to target cell populations, caused changes consistent with the obese phenotype (10).

As an important transmitter of cellular information, exosomes have received extensive attention in recent years. The potential role of exosomes in intercellular communication makes exosomes considered an important endocrine mechanism. Current issues surrounding exosome biogenesis mainly focus on the components contained in exosomes and the effects of exosomes on recipient cells (11). The size, content, origin, etc. of exosomes all contribute to exosome heterogeneity (12). Exosomes derived from different tissues and cells contain different properties, which may cause uptake by some specific cells or organs or have different effects on different recipient cells (13). This heterogeneity adds complexity to the function of exosomes in cell-to-cell and organ-to-organ communication. Adipose tissue, as an important endocrine organ, plays an indispensable role in intercellular communication, interorgan communication and systemic metabolic homeostasis. Therefore, elucidating the role of specific tissue-derived exosomes (such as adipose tissue-derived exosomes) in the body can provide very deep evidence for their further application and clinical translation (10).

This review focuses on the role of adipose tissue-derived exosomes in different metabolic diseases. We have introduced the effect of adipose tissue-derived exosomes on homeostasis and the molecular mechanism, and summarized the research status of adipose tissue-derived exosomes in stem cell therapy. Also, we have reviewed some effects of dysregulated metabolic homeostasis on exosomes.

## Biogenesis, Composition and Isolation of Exosomes

### Biogenesis of Exosomes

**Figure 1:**
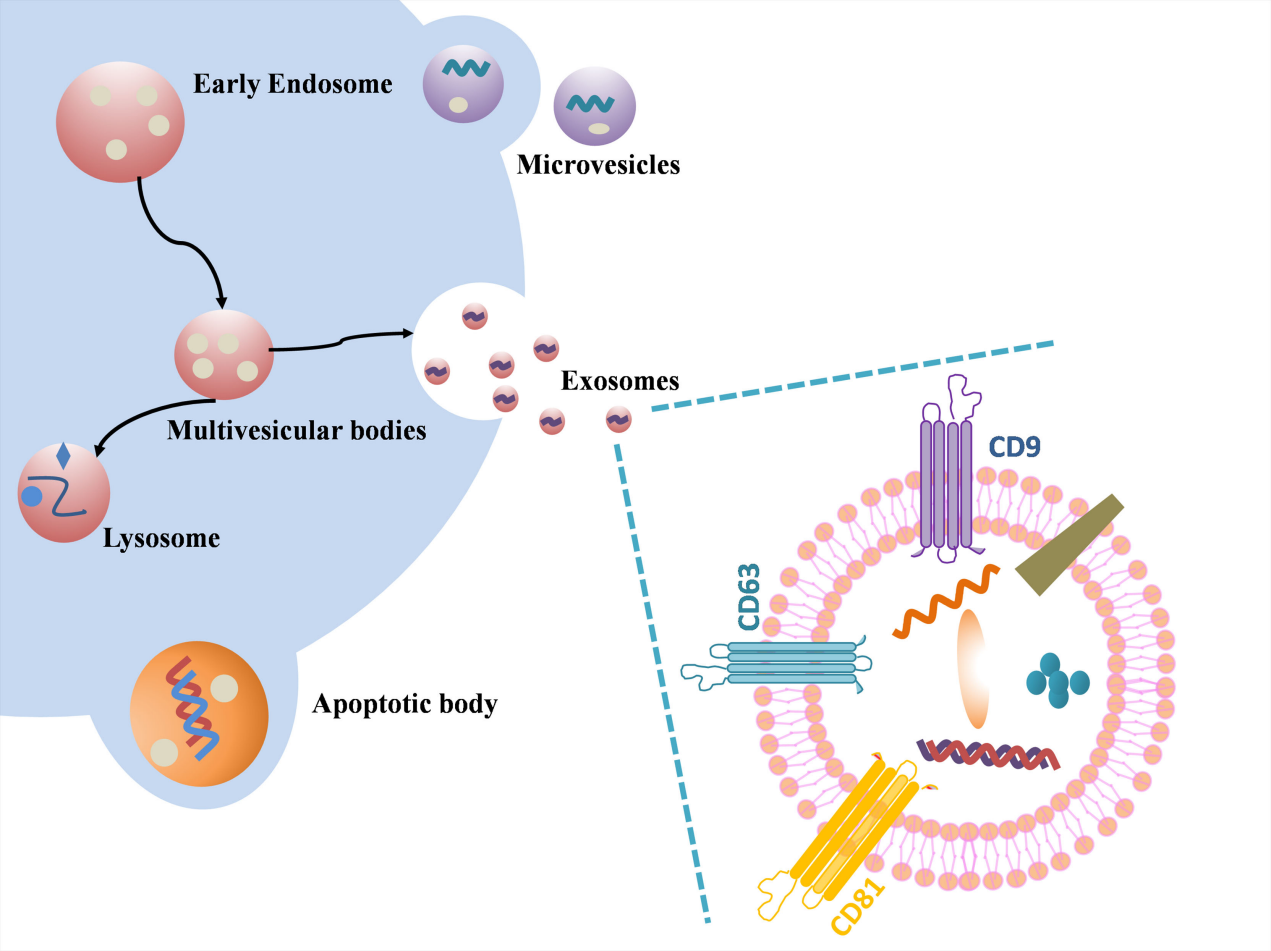
Schematic illustration of exosome biogenesis and component. Differently from microvesicular bodies (MVBs) and apoptotic bodies, exosome derive from the endosomal pathway by evolution of the early and then late endosomes, and ultimately MVBs fuse with plasma membrane to release the exosomes. Exosome surface proteins include characteristic markers (CD9, CD81, CD63 and so on). Exosomes encapsulate various sorts of cell surface and intracellular proteins, coding and non-coding RNAs, DNA, amino acids, and some metabolites.

Extracellular vesicles are a general term for a variety of nano-scale vesicles that are actively released by cells, which can be divided into microvesicles budding from the plasma membrane, apoptotic bodies shed from dying and disintegrating cells, and exosomes derived from the endolysosomal pathway (14–16) (**Figure 1**). Plasma membrane funnel-like internalization of cell surface proteins and soluble proteins associated with the extracellular environment led to the formation of early endosomes (17, 18). Incoming endocytic cargo matures into late endosomes through clathrin-dependent or clathrin-independent pathways and ultimately forms MVBs. This process is accompanied by the recruitment of partially soluble molecules (cytosolic proteins and RNA) into ILVs(intraluminal vesicles) (19–21). Different MVBs have different fates and can either fuse with lysosomes or autophagosomes to be degraded, or fuse with the plasma membrane of the parent cell to release the contained ILVs as exosomes (17, 22, 23). Rab family (such as Rab27A, Rab27B) are key mediators of exosome release (23), SNARE (soluble N- ethylmaleimide- sensitive factor attachment protein receptor) can drive membrane fusion to promote exosome secretion (24). The exosomes released outside the cell can fuse with the receptor cytoplasmic membrane to release the cargo directly, or the exosomal transmembrane protein can directly interact with the signaling receptor of the target cell (25–27). The third way in which exosomes released into the extracellular space communicate with recipient cells is that exosomes enter recipient cells by phagocytosis/endocytosis (28, 29), some phagocytosed exosomes will merge into endosomes by endocytosis and be released into adjacent cells, and others will mature into lysosomes and degrade after fusion with endosomes (30).

Tetraspanins, ESCRT (endosomal sorting complex required for transport) protein, phospholipids, TSG101 (tumor susceptibility gene 101), ALIX (ALG-2-interacting protein X), ceramide and SNARE, etc. are involved in exosome biogenesis. Tetraspanins are not only one of the most commonly used exosome marker proteins, but also promote the transport of other membrane proteins and improve their stability (19). As an exosomal scaffold protein, Alix binds TSG101 and ESCRT. Studies have shown that the ESCRT-related cytoplasmic protein ALIX is not only recruited to endosomes through its interaction with LBPA (lysobisphosphatidic acid) (22), but also regulates the formation of ILVs in acidic late endosomes and thus affects exosome generation (27). Trajkovic et al. found that purified exosomes were rich in ceramides, and inhibition of neutral sphingomyelinase reduced exosome release (31). Ceramides can stimulate exosome production and the content of ceramides is positively correlated with the increase of exosome biosynthesis.

### Composition of Exosomes

The size of exosomes is arranged from 40nm to 160 nm. Exosomes contain mRNA, microRNA (miRNA), ribosomal RNA, long non-coding RNA (lncRNA) and DNA, proteins and lipids (32). Exosomes from different sources contain different structural proteins and lipids. Although exosomes are formed by the invagination of the plasma membrane, the exosome membrane also includes lipids from the Golgi complex resulting in a different membrane lipid content than the plasma membrane (21). The most common proteins include tetraspanins (like CD63, CD9, CD81), heat shock proteins (like Hsp70, Hsp90) and endosomal markers (like Alix). It is worth noting that in addition to the rest of the common cytoskeletal proteins, albumin, etc., major histocompatibility complex class I (MHC I) is expressed on all exosomes, while major histocompatibility complex class II (MHC II) is only expressed on antigen-presenting cells derived exosomes (33). Microvesicles and apoptotic bodies are extracellular vesicles of 50nm -5μm and 1μm -5μm in size, respectively. Microvesicles were originally studied for their role in blood coagulation and were formerly known as “platelet dust” (34). Unlike exosomes, microvesicle biogenesis involves changes in lipid composition, protein composition, and Ca2^+^ levels. Microvesicles contain a large amount of cholesterol and calpain, which facilitates membrane budding and the formation of microvesicles (35). Compared to exosomes, microvesicles, apoptotic bodies are relatively large extracellular vesicles that contain remnants of encapsulated dying cells. Phosphatidylserine is the only marker of apoptotic bodies found so far (36). Recent studies on apoptotic bodies have found that apoptotic bodies can not only activate the immune system, but also transmit genetic information. Samos et al. detected apoptotic bodies in the blood of tumor xenograft-bearing mice (37). Due to cross-running of exosomes with the biogenesis pathways of the remaining extracellular vesicles, the precise rate-limiting roles and functions of these molecules in exosome biogenesis need to be further explored.

## Role of Adipose Tissue-Derived Exosomes in Metabolic Diseases

**Figure 2:**
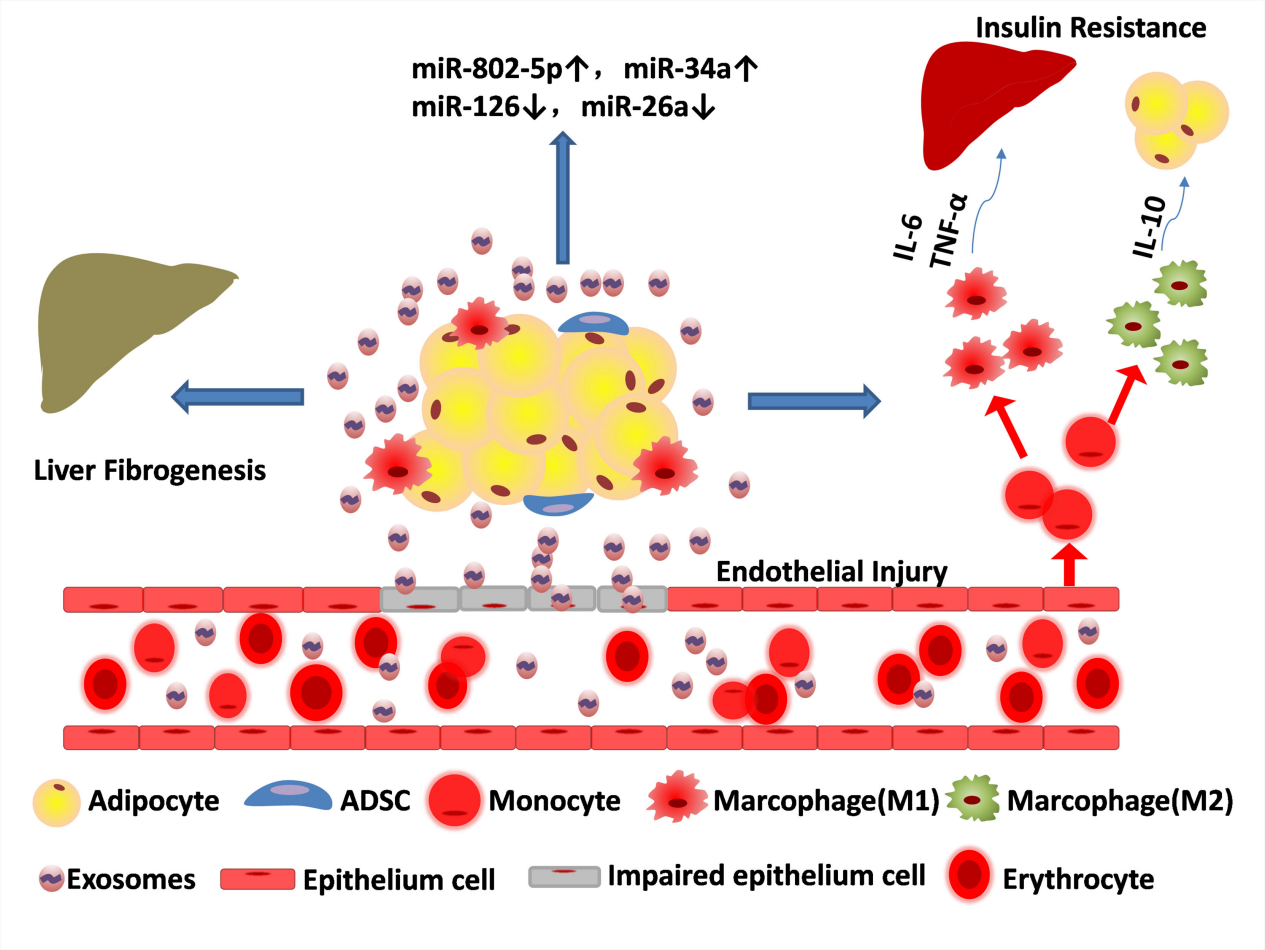
Adipose tissue-derived exosomes result in metabolic disorders. Exosomes released from adipose tissue can destroy the epithelium cell, ruin the blood vessels, aggravate liver fibrogenesis and promote transformation of monocyte to both macrophage (M1 pro-inflammatory phenotype) and macrophage (M2 anti-inflammatory phenotype). Cytokines such as IL-6 and TNF-α released by M1 can affect systemic metabolism and exacerbate insulin resistance, while IL-10 released by M2 can alleviate adipose inflammation. The content of miRNAs in adipose tissue-derived exosomes are partially upregulated (e.g. miR-802-5p, miR-34a) and some are down regulated (miR-126, miR-26a).

In addition to regulating whole-body energy metabolism through adipocytes, adipose tissue also produces a variety of adipokines, including leptin and adiponectin (38). These adipokines play a role not only in maintaining glucose, lipid and energy homeostasis, but also in communication between adipose tissue or between adipose tissue and other tissues (39). Adiponectin is a pleiotropic organ-protective protein secreted only by adipocytes (40). T-cadherin promotes the accumulation of adiponectin in multivesicular bodies to stimulate exosome biogenesis and secretion (40). Wei et al. found that adipose tissue-derived exosomes were able to modulate insulin sensitivity *in vitro* and *in vivo (* 41). Therefore, exosomes not only affects the process of metabolism, but in turn there biogenesis is regulated by metabolism. In addition to adipocytes and adipokines, adipose tissue-derived exosomes, as an emerging cell-to-cell and organ-to-organ communicator, play a role in metabolic homeostasis worthy of in-depth study (**Figure 2**).

### Obesity

The most important feature of obesity is the excessive accumulation of adipose tissue (42). There is increasing evidence that adipose tissue-derived exosomes not only function in paracrine and endocrine modes, but also act as intercellular communicators to facilitate the transition of adipocytes towards maturity (43). In studies of the role of exosomes in systemic metabolism, adipocyte-derived exosomes were found to be mediators linking obesity and insulin resistance in surrounding tissues such as the liver, interacting with adjacent sites to promote lipid esterification (44, 45). Connolly et al. showed that exosomes per adipocyte have higher yields in the preadipocyte stage and are rich in pre-signal fatty acids (46). Exosomes contain different fatty acids with different stages of differentiation. Interestingly, ATM (adipose tissue macrophage)-derived exosomes not only modulate adipose tissue function and insulin sensitivity, but also promote the activation of monocytes to macrophages (M1 pro-inflammatory phenotype) after uptake by peripheral blood (47). Wei et al. found that miRNAs contained in adipose tissue macrophage-derived exosomes from obese mice promoted insulin resistance, while ATM-derived exosomes from lean mice attenuated insulin resistance in obese mice (48). In addition, it has been demonstrated obesity has an impact on the cargo carried by adipocyte-derived exosomes. Carlos et al. used exosomes from obese mice to treat lean mice with glucose intolerance and insulin resistance, and further found that obesity alters the distribution of exosomal miRNAs in mice (49). Studies on the 3T3-L1 adipocyte model showed that these adipocytes increased the content of exosomal miR-802-5p, which promotes insulin resistance in cardiomyocytes by downregulating HSP60 (50). Clinical studies have shown that subcutaneous adipocyte-derived exosomes from obese patients are enriched in proteins related to fatty acid oxidation, leading to pathophysiological changes (51). Although many researches have showed that macrophages in adipose tissue are the main pool of exosomes, new studies have showed that it is adipocytes that released large quantities of exosomes.

The effect of adipocyte-derived exosomes on body metabolism has been widely recognized. However, the functional impact of adipocyte-derived exosomes on the control of adipogenesis needs to be further explored to clarify its profound impact in cardiovascular and metabolic diseases. On the other hand, the effect of obesity on the cargo contained in adipocyte-derived exosomes has been intensively studied and explored (52). However, since there is no specific cargo selection process for cellular uptake of exosomes, the relationship between the effect of exosomes on recipient cells and specific exosome contents needs to be further clarified to facilitate design exosomes for disease treatment.

### Diabetes

Diabetes is a group of metabolic disorders characterized by chronically high levels of blood sugar due to insufficient insulin production (T1DM) or poor response of receptor cells to insulin (T2DM) (4, 10). Exosomes have recently been investigated as a potential candidate for biomarkers involved in obesity-related diabetes progression and prognosis. Existing evidence suggests that adipose tissue may coordinate systemic metabolic homeostasis through exosomes, but the quantity, quality, and cargo of exosomes produced by adipose tissue are abnormal under pathological conditions (26, 53). Adipose tissue is divided into white adipose tissue (WAT) and brown adipose tissue (BAT). The number of exosomes extracted from white adipose tissue in patients with inflammation and systemic insulin resistance is increased, the uptake of exosomes by leukocytes is increased, and the cargo carried by exosomes may signal the conversion of endothelial cells with a normal phenotype to a diabetic phenotype (54). Clair et al. found that adipose tissue-derived exosomes were more responsive to glucagon compared to the exosomes derived from other tissues (55). Glucagon not only accelerated the uptake of BSA and cargo into exosomes by adipose tissue endothelial cells, but also enhanced the secretion of exosomes (56, 57). Because endothelial cell exosomes contain extracellular/serum signaling molecules, it can be speculated that the type of signaling in the blood can affect endothelial cell exosomes and produce functional responses to adipocytes and metabolic systems (58, 59).

Adipocyte-derived exosomes and adipose tissue macrophage-derived exosomes drive this intra-organ crosstalk of adipocytes and macrophages in type 2 diabetes. In type 2 diabetes, sonic hedgehog containing exosomes can mediate pro-inflammatory macrophage (M1 pro-inflammatory phenotype) activation (60). Macrophages (M1 pro-inflammatory phenotype) affect the metabolic status of adipocytes by releasing cytokines (like IL-6, TNF-α), leading to systemic glucose intolerance and insulin resistance (61, 62). Ying et al. found that injection of ATM (Adipose Tissue Macrophage)-derived exosomes from obese mice into lean mice reduced the expression levels of peroxisome proliferator-activated receptor γ (PPARγ), glucose transporter type 4 (GLUT4) and adipocyte sensitivity to insulin (41, 63). miRNA-155, an inhibitor of PPARγ, has been shown to be the most critical factor in ATM exosomes affecting metabolic disorders (64). Surprisingly, in one study, brown adipocyte-derived exosomes from normal mice alleviated systemic insulin resistance in ADiceKO mice after being taken up by the liver (65). Existing evidence suggest that adipose tissue, especially brown adipose tissue-derived exosomes, maintain metabolic homeostasis under physiological conditions (66, 67), while under pathological conditions, such as T2DM, adipose tissue-derived exosomes cause intra-organ crosstalk synergy to aggravate glucose intolerance and insulin resistance (68, 69).

### Adipose Inflammation

In the progression of obesity, chronic overnutrition often causes unhealthy expansion of adipose tissue leading to immune cell infiltration, which in turn triggers acute and chronic inflammation of the tissue (70, 71). The most important factor causing a series of inflammation is the inflammation of adipose tissue. Macrophages are the main immune cells in adipose tissue and exhibit different phenotypes depending on the changing environment in the body (72). ATM undergoes a transition from an anti-inflammatory M2 phenotype to an M1 pro-inflammatory phenotype during the progression of obesity and produces pro-inflammatory factors to exacerbate adipose inflammation (73). In addition to macrophages, obese adipose tissue recruits additional immune cells. Studies have shown that CD4+ and CD8+ T cells secreting proinflammatory mediators are increased in WAT (74). Studies have shown that injection of M2 macrophage-derived exosomes into obese mice can significantly improve insulin-glucose homeostasis (75, 76). The exosomes secreted by adipocytes carrying miRNA-34a inhibited the polarization of M2 macrophages to promote the occurrence and development of adipose inflammation, and further found that miRNA-34a KO mice had higher levels of adiponectin than WT mice (77). Adiponectin, an adipokine with insulin-sensitizing activity, abolished macrophage-involved extracellular matrix remodeling, such as collagen formation and fibrosis (78). Adiponectin, Omentin-1 and Secreted Frizzled-Related Protein 5 (SFRP5) are anti-inflammatory adipokines (1, 79, 80). Omentin-1 has anti-inflammatory, anti-obesity, and anti-diabetic properties (81, 82). Omentin-1 enhances insulin stimulation to enhance glucose uptake and activate insulin receptor substrate (IRS) by inhibiting mTOR signaling pathway (83, 84). SFRP5 is thought to be a negative regulator of adipose tissue-related chronic inflammation. SFRP5 inhibits Wnt5a-mediated inflammation, obesity and insulin resistance by binding to Wnt proteins (85). Serum SFRP5 levels were lower in obese and T2DM patients, and SFRP5 depletion increased macrophage numbers and pro-inflammatory proteins in mouse adipose tissue (86–88).

## Effects of Adipose Tissue-Derived Exosomal Cargo on Metabolic Diseases

miRNAs are non-coding RNA molecules that mediate post-transcriptional gene silencing by binding to the 3 ‘-untranslated region (UTR) or open reading frame (ORF) region of target gene mRNAs (89). In the nucleus, RNA polymerase II transcribes primary miRNAs (pri miRNAs) and is exported to the cytoplasm upon processing by the endoribonuclease DROSHA and its RNA-binding partners (90, 91). The miRNA duplex is then produced by further processing of the type III endoribonuclease DICER with RNA-binding proteins (92). Eventually the double-stranded miRNA is loaded into the RNA-induced silencing complex (RISC), and Argonaute-2 (AGO2) and chaperones guide it to interact with target mRNAs (93).

### Potential Mechanisms by Which miRNAs Are Sorted Into Exosomes

miRNA can be packaged into exosomes, loaded into high-density lipoprotein (HDL), and bound to AGO2 protein outside the vesicle to avoid miRNA degradation (94). The proportion of miRNAs in exosomes is higher than in parental cells. Studies have confirmed that miRNAs do not enter exosomes randomly, and some miRNAs (e.g. miRNA-150, miRNA-451) preferentially enter the exosome lumen (95). Several studies have shown that AGO2 and other RNA-binding proteins are involved in regulating the process of miRNA entry into exosomes (94). According to current researches, several potential approaches have been put forward to show the exosomal miRNA sorting modes. In 2013s, Kosaka et al. firstly found that overexpression of sphingomyelinase 2 (nSMase2) in cancer cells can increase the number of exosomal miRNAs and promote angiogenesis as well as metastasis in tumor microenvironment (96). miRNA motif and sumoylated heterogeneous nuclear ribonucleoproteins (hnRNPs)-dependent pathway have also been reported to regulate exosomal miRNA sorting. Both Villarroya Beltri and Gao et al. have found that hnRNPA2B1 could recognize the GGAG motif in the 3’ sides of miRNA and result in specific miRNAs to be packed into exosomes (97, 98). Similarly, Koppers-Lalic et al. have revealed that 3’ sides of uridylated endogenous miRNAs were mainly sorted in exosomes derived from B cells or urine, in the meanwhile, the 3’ sides of adenylated endogenous miRNAs were mainly concentrated in B cells (99). Also, recent studies have found that miRNA-induced silencing complex (miRISC)-related pathway might be correlated with exomal miRNA sorting (100). For instance, Guduric Fuchs et al. have discovered that knockout of AGO2 in 293T cells can downregulate the bundance of the specific miRNAs such as miR-451 and miR-142-3p in exosomes (95). In the past year, Xiao-Man Liu et al. have found that phase separation-mediated local enrichment of cytosolic RNA-binding proteins enables their targeting and packaging by exosomes (101). They found that YBX1 protein efficiently forms liquid-like droplets in cells and miR-223 could be efficiently partitioned into YBX1 droplets (101). Although miRNA sorting to exosomes might be a passive mechanism to deliver miRNAs even in excess of their acceptor cells, emerging researches suggest that exosomes can be actively uptake by other cells. These results lead to specific miRNA transfer among different cells. In addition, certain miRNA signatures in exosomes might represent biomarkers of diseases. For example, tumor cells have altered transcriptomic profiles, which in turn will significantly influence miRNA sorting to exosomes (102). A great number of previous reviews have summarized the exosomal miRNA can act as biomarkers. Not only miRNA can function as biomarkers, but also miRNAs can reach neighboring and distant cells through exosome circulation, mediate cell-to-cell communication by targeting mRNAs and confer characteristic changes in the expression levels of target genes (103). Thomas Thomou ei al. have found that adipose tissues are major source of circulating exosomal miRNAs and function as gene regulator in distant tissues thereby serving as a novel adipokine (104). To be specific, they found that mice with adipose-specific knockout of the miRNA-processing enzyme Dicer (ADicerKO), as well as humans with lipodystrophy, have less circulating exosomal miRNAs compared with normal samples (104). Similarly, C. Ronald Kahn et al. also have found that different cell types released different amounts of exosomes and differentiated 3T3-L1 cells (white adipocytes) having the highest production and release rates per cell (102). Furthermore, they found that miRNAs possess sorting sequences which determine their secretion in exosomes or cellular retention and that different cell types make preferential use of specific sorting sequences, thus defining the exosomal miRNA profile of that cell type (105).

### Impact of Adipose Exosomal miRNAs on the Metabolism

Adipose tissue is a particularly important contributor to the circulating exosomal miRNA pool, with adipocytes and stem cells expressing a wide range of miRNAs. miRNAs in adipose tissue-derived exosomes promote metabolic homeostasis in an endocrine manner. Thomas et al. used ADicerKO mice to significantly reduce exosomal miRNAs, reduce WAT, and whiten BAT (104). After transplantation of adipose tissue into ADicerKO mice, the glucose tolerance of the mice improved, and the content of most exosomal miRNAs returned to normal levels (104). Interestingly, serum FGF21 (fibroblast growth factor-21) and liver FGF21 mRNA remained unchanged in ADicerKO mice after WAT transplantation (104). In contrast, FGF21 mRNA in the liver of ADicerKO mice transplanted with BAT was reduced by about 50% (104). To further clarify which miRNAs might regulate FGF21, AML-12 hepatocytes were transfected with adenoviral pacAd5-FGF213’-UTR luciferase reporter gene and found that only miR-99b resulted in a significant decrease in FGF21 luciferase activity (104). Other studies have shown that miRNAs in adipose tissue-derived exosomes may also regulate body metabolism in a paracrine manner. Exosomes containing miR-16, miR-27a, miR-146b and miR-222 released from adipocytes can enter small adipocytes to stimulate adipogenesis (49). By comparing exosomes released by adipose tissue-derived stem cells (ADSC-Exos) with exosomes released by adipose tissue (AT-Exos), Zhang et al. found that compared with ADSC-Exos, AT-Exos contained 7 times more miR-450a, While miR-450a increases adipogenesis by inhibiting WISP2 (106). This study demonstrated that exosomal miRNAs also have autocrine functions. Adipose tissue macrophage-derived exosomes also overexpressed miR-155. miR-155 inhibits insulin action by downregulating PPARγ mRNA (41, 65). Diabetic patients had lower levels of miRNA-126 and miRNA-26a compared to normal individuals, which are characteristic markers of endothelial cell-derived exosomes (107, 108). Studies have shown that the release of exosomes is up-regulated by physiological (such as insulin) and pharmacological (such as glimepiride) stimuli (109). Fang et al. reported that after administration of rosiglitazone, adipocytes secreted exosomes containing a large amount of miR-200 and were taken up by cardiomyocytes resulting in hypertrophic changes (110). Interorgan crosstalk mediated by exosomal miR-200 may be the molecular mechanism of the adverse effects of rosiglitazone. However, studies on the effects of metabolized drugs on the production, release, and transport of exosomes on the body are limited, but research to explore the therapeutic application of exosomes still holds attractive prospects. In addition to adipose tissue-derived exosomal miRNAs, circulating miRNAs from other sources have increasingly been found to be strongly linked to metabolic disorders (111). Continued in-depth exploration of miRNAs will expand our understanding of the specific relationship between miRNAs and body metabolism.

### Proteins and Other Cargoes

Besides miRNAs, adipose tissue-derived exosomes or other cargoes are also involved in metabolic homeostasis. Liu et al. transferred adipose tissue-derived exosomes to mouse macrophages by melatonin to reduce metabolic inflammation and increase α-ketoglutarate (αKG) levels, and found that αKG is a melatonin inhibitor of adipose inflammation ‘s target (112). Melatonin attenuates adipocyte inflammation by promoting TET-mediated DNA methylation by transporting adipose tissue-derived exosomal αKG to macrophages (112). Studies have isolated exosomes derived from adipose-derived mesenchymal stem cells, and demonstrated that glyoxalase-1 (GLO-1) is highly expressed in the exosomes and efficiently targeted to deliver adipose-derived exosomes loaded with GLO-1 protein bygenetic engineering to protect endothelial cells and enhance angiogenesis in type 2 diabetic mice (113). Regarding the study of proteins and other cargoes, current evidence suggests that adipose-derived exosomes play an active role in metabolic homeostasis.

## Therapeutic Potential of ADSCs-Exos in Metabolic Diseases

Mesenchymal stem cells (MSCs) in the stromal vascular part of adipose tissue, also known as adipose-derived stem cells (ADSCs), have the ability to differentiate into pluripotent cells (114). ADSCs can produce and secrete a large number of exosomes (ADSCs-Exos), which inherit many functions of cells, such as immune regulation, angiogenesis, proliferation and migration (115). Notably, miRNAs (e.g. miR-126, miR-130a, miR-132) carried by ADSCs-Exos were able to increase growth factors (like epidermal growth factor, fibroblast growth factor) in endothelial cells to promote angiogenesis (116, 117). Han et al. further found that hypoxia-treated ADSCs-Exos enriched more growth factors and had higher pro-angiogenic capacity. There is increasing evidence that ADSCs-derived exosomes (ADSC-Exos) can transfer DNA, RNA and proteins to adjacent cells or tissues (118). ADSCs-Exos not only reduced the secretion of IFN-γ to inhibit T cell activation to reduce adipose inflammation and immune responses, but also dominated the polarization of the anti-inflammatory (M2) macrophage phenotype, which can express high levels of tyrosine Hydroxylase, thereby releasing catecholamines, activates the expression of white adipose tissue (WAT)-specific uncoupling protein 1, remodeling immune homeostasis in WAT (119, 120). Another study reported that ADSCs-Exos from epididymal WAT polarized macrophages to an M2 phenotype by transferring active STAT3, further promoting browning of WAT and reducing adipose inflammation (119). Nevertheless, there are still few studies on the relationship between ADSC-Exos and metabolism. Some studies even shown that when adipose tissue-derived exosomes and ADSCs-Exos were respectively co-cultured with ADSCs, only adipose tissue-derived exosomes could induce adipogenesis, ADSC-Exos could not (121, 122). However, its specific molecular mechanism is still unclear. More attention and research are still needed on the role of exosomes as adipogenic molecules on the adipose tissue microenvironment and the interaction between adipose-derived mesenchymal stem cells and adipose tissue cells in the microenvironment. Although there are some limitations of ADSC-Exos, ADSC-Exos is still a rising star in cell-free therapeutic drugs, and even has the potential to become an alternative to ADSC (123). Li et al. demonstrated the ability to promote skin healing in rats with diabetic foot ulcers by using ADSC-Exos overexpressing Nrf2 (124). Shilan et al. transplanted ADSC-Exos-loaded alginate hydrogel into the full-thickness wound excision site of rats to promote tissue regeneration of rat skin wounds (125). Encouragingly, related studies have demonstrated that preservation of exosomes with trehalose, a natural nontoxic cryoprotectant, can prevent exosome aggregation and cryo-damage compared to PBS (126). We have every reason to believe that ADSC-Exos is a very promising regenerative therapy.

## Conclusion and Future Perspectives

In the past, the understanding of metabolic diseases mainly relied on the examination of biochemical indicators and physical signs. The impact of adipose tissue-derived exosomes on metabolic disorders such as obesity is emerging. Exosomes are endogenous products, thus understanding the biogenesis, transfer, and molecular mechanisms of exosomes causing metabolic disorders in the body will help to design new therapies for various metabolic diseases mediated by adipose tissue-derived exosomes (17). However, current researches on exosomes still lack breakthrough progress, especially the methods for isolating specific types of exosomes are still immature. The current extraction methods of exosomes mainly include: A. Differential ultracentrifugation, which is convenient to operate but has the disadvantage of relatively less yield and protein and RNA contamination. B. Density gradient ultracentrifugation, which is efficient to separate exosomes but is time consuming and low yield. C. Polymer precipitation, which is easy and time-saving but not pure. D. Size exclusion chromatography, which has high purity and reproducibility but time-consuming. E. Immunoaffinity, which is suitable for isolating specific exosomes but not consuming. F. Microfluidics-based isolation, which has high purity but low yield. Based on the advantages and disadvantages of the above methods, as well as previous studies, we believe that it is crucial to develop new and efficient methods of exosome extraction, so exploring the biogenesis and other related properties of exosomes is an important prerequisite for developing new methods. Although proteomics of exosomes revealed the heterogeneity of exosome markers, it suggested that exosomes could be purified according to different markers, which improved the practicability of exosome research and experimental design, and promoted the research progress of exosomes from different tissue (127, 128). However, exosome size heterogeneity, content heterogeneity, functional heterogeneity, and labeling heterogeneity limit the study of exosome-mediated processes (129). Studying the content of exosomes by transient stimulation, the transient changes in the content of the contained cargo to deeply explore cellular functions may be a feasible means to understand the different mechanisms and scope of exosome action.

In this review, we elaborate on the biogenesis of exosomes and the roles that adipose tissue exosomes play in metabolic diseases. At present, the research on adipose tissue-derived exosomes mainly focuses on the research of the cargo (miRNA, protein, etc.) contained in the exosomes. Growing evidence suggests that obesity-related and adipose tissue-derived miRNAs hold promise as novel therapeutic targets for the treatment of obesity and related diseases (130). Key aspects such as the underlying mechanisms of exosome-mediated crosstalk in the body’s metabolic environment, long-range cellular interactions, and heterogeneity of exosomes have been intensively studied. These studies are beneficial to the development of new exosome-based therapeutics, such as miRNA mimics, miRNA-loaded exosomes and other gene therapies may have a very promising market and future in metabolic diseases. However, few studies have combined the RNA contained in exosomes with proteomics to synergistically study its impact on the body’s metabolism. Combining the two studies may provide new directions and insights for exosomes in the diagnosis and treatment of diseases. There is still a long way to go to overcome the current obstacles hindering exosome-based therapeutic strategies for metabolic diseases.

## Author Contributions

ZW and LL designed the structure and conceived the manuscript. RM, WQ, and YZ wrote the manuscript. All authors contributed to the article and approved the submitted version.

## Funding

This work was supported by General development projects of Shaanxi Province (2018YBXM-SF-12-2 to WQ).

## Conflict of Interest

The authors declare that the research was conducted in the absence of any commercial or financial relationships that could be construed as a potential conflict of interest.

## Publisher’s Note

All claims expressed in this article are solely those of the authors and do not necessarily represent those of their affiliated organizations, or those of the publisher, the editors and the reviewers. Any product that may be evaluated in this article, or claim that may be made by its manufacturer, is not guaranteed or endorsed by the publisher.

## References

[1] Lee M-W Lee M Oh K-J. Adipose Tissue-Derived Signatures for Obesity and Type 2 Diabetes: Adipokines, Batokines and MicroRNAs. J Clin Med (2019) 8(6):854. doi: 10.3390/jcm8060854 PMC6617388 31208019

[2] Sun K Tordjman J Clement K Scherer PE. Fibrosis and Adipose Tissue Dysfunction. Cell Metab (2013) 18(4):470–7. doi: 10.1016/j.cmet.2013.06.016 PMC3795900 23954640

[3] Unamuno X Gomez-Ambrosi J Rodriguez A Becerril S Fruhbeck G Catalan V. Adipokine Dysregulation and Adipose Tissue Inflammation in Human Obesity. Eur J Clin Invest (2018) 48(9):e12997. doi: 10.1111/eci.12997 29995306

[4] Burhans MS Hagman DK Kuzma JN Schmidt KA Kratz M. Contribution of Adipose Tissue Inflammation to the Development of Type 2 Diabetes Mellitus. Compr Physiol (2019) 9(1):1–58. doi: 10.1002/cphy.c170040 PMC6557583 30549014

[5] Goodpaster BH Sparks LM. Metabolic Flexibility in Health and Disease. Cell Metab (2017) 25(5):1027–36. doi: 10.1016/j.cmet.2017.04.015 PMC5513193 28467922

[6] Heindel JJ Blumberg B Cave M Machtinger R Mantovani A Mendez MA. Metabolism Disrupting Chemicals and Metabolic Disorders. Reprod Toxicol (2017) 68:3–33. doi: 10.1016/j.reprotox.2016.10.001 27760374 PMC5365353

[7] Hartwig S De Filippo E Goeddeke S Knebel B Kotzka J Al-Hasani H. Exosomal Proteins Constitute an Essential Part of the Human Adipose Tissue Secretome. Biochim Et Biophys Acta-Proteins Proteomics (2019) 1867(12):140172. doi: 10.1016/j.bbapap.2018.11.009 30502511

[8] Hu W Ru Z Xiao W Xiong Z Wang C Yuan C. Adipose Tissue Browning in Cancer-Associated Cachexia can be Attenuated by Inhibition of Exosome Generation. Biochem Biophys Res Commun (2018) 506(1):122–9. doi: 10.1016/j.bbrc.2018.09.139 30340833

[9] Huang Z Xu A. Adipose Extracellular Vesicles in Intercellular and Inter-Organ Crosstalk in Metabolic Health and Diseases. Front Immunol (2021) 12. doi: 10.3389/fimmu.2021.608680 PMC7946830 33717092

[10] Chait A den Hartigh LJ. Adipose Tissue Distribution, Inflammation and Its Metabolic Consequences, Including Diabetes and Cardiovascular Disease. Front Cardiovasc Med (2020) 7. doi: 10.3389/fcvm.2020.00022 PMC7052117 32158768

[11] Das CK Jena BC Banerjee I Das S Parekh A Bhutia SK. Exosome as a Novel Shuttle for Delivery of Therapeutics Across Biological Barriers. Mol Pharmaceut (2019) 16(1):24–40. doi: 10.1021/acs.molpharmaceut.8b00901 30513203

[12] Lai RC Yeo RWY Tan KH Lim SK. Exosomes for Drug Delivery - a Novel Application for the Mesenchymal Stem Cell. Biotechnol Adv (2013) 31(5):543–51. doi: 10.1016/j.biotechadv.2012.08.008 22959595

[13] Lai RC Chen TS Lim SK. Mesenchymal Stem Cell Exosome: A Novel Stem Cell-Based Therapy for Cardiovascular Disease. Regener Med (2011) 6(4):481–92. doi: 10.2217/rme.11.35 21749206

[14] Bebelman MP Smit MJ Pegtel DM Baglio SR. Biogenesis and Function of Extracellular Vesicles in Cancer. Pharmacol Ther (2018) 188:1–11. doi: 10.1016/j.pharmthera.2018.02.013 29476772

[15] Colombo M Raposo G Thery C. Biogenesis, Secretion, and Intercellular Interactions of Exosomes and Other Extracellular Vesicles. Annu Rev Cell Dev Biol (2014) 30:255–89. doi: 10.1146/annurev-cellbio-101512-122326 25288114

[16] Doyle LM Wang MZ. Overview of Extracellular Vesicles, Their Origin, Composition, Purpose, and Methods for Exosome Isolation and Analysis. Cells (2019) 8(7):727. doi: 10.3390/cells8070727 PMC6678302 31311206

[17] Kalluri R LeBleu VS. The Biology, Function, and Biomedical Applications of Exosomes. Science (2020) 367(6478):eaau6977. doi: 10.1126/science.aau6977 32029601 PMC7717626

[18] Mathieu M Martin-Jaular L Lavieu G Thery C. Specificities of Secretion and Uptake of Exosomes and Other Extracellular Vesicles for Cell-to-Cell Communication. Nat Cell Biol (2019) 21(1):9–17. doi: 10.1038/s41556-018-0250-9 30602770

[19] Farooqi AA Desai NN Qureshi MZ Nogueira Librelotto DR Gasparri ML Bishayee A. Exosome Biogenesis, Bioactivities and Functions as New Delivery Systems of Natural Compounds. Biotechnol Adv (2018) 36(1):328–34. doi: 10.1016/j.biotechadv.2017.12.010 29248680

[20] Ferguson SW Nguyen J. Exosomes as Therapeutics: The Implications of Molecular Composition and Exosomal Heterogeneity. J Contro Release (2016) 228:179–90. doi: 10.1016/j.jconrel.2016.02.037 26941033

[21] Gurunathan S Kang M-H Kim J-H. A Comprehensive Review on Factors Influences Biogenesis, Functions, Therapeutic and Clinical Implications of Exosomes. Int J Nanomed (2021) 16:1281–312. doi: 10.2147/IJN.S291956 PMC7898217 33628021

[22] Gurung S Perocheau D Touramanidou L Baruteau J. The Exosome Journey: From Biogenesis to Uptake and Intracellular Signalling. Cell Commun Signal (2021) 19(1):47. doi: 10.1186/s12964-021-00730-1 33892745 PMC8063428

[23] Hessvik NP Llorente A. Current Knowledge on Exosome Biogenesis and Release. Cell Mol Life Sci (2018) 75(2):193–208. doi: 10.1007/s00018-017-2595-9 28733901 PMC5756260

[24] Kim H Kim EH Kwak G Chi S-G Kim SH Yang Y. Exosomes: Cell-Derived Nanoplatforms for the Delivery of Cancer Therapeutics. Int J Mol Sci (2021) 22(1):14. doi: 10.3390/ijms22010014 PMC7792591 33374978

[25] Zhang H Freitas D Kim HS Fabijanic K Li Z Chen H. Identification of Distinct Nanoparticles and Subsets of Extracellular Vesicles by Asymmetric Flow Field-Flow Fractionation. Nat Cell Biol (2018) 20(3):332–43. doi: 10.1038/s41556-018-0040-4 PMC5931706 29459780

[26] Yang B Chen Y Shi J. Exosome Biochemistry and Advanced Nanotechnology for Next-Generation Theranostic Platforms. Adv Mater (2019) 31(2):e1802896. doi: 10.1002/adma.201802896 30126052

[27] Yang D Zhang W Zhang H Zhang F Chen L Ma L. Progress, Opportunity, and Perspective on Exosome Isolation - Efforts for Efficient Exosome-Based Theranostics. Theranostics (2020) 10(8):3684–707. doi: 10.7150/thno.41580 PMC7069071 32206116

[28] Barile L Vassalli G. Exosomes: Therapy Delivery Tools and Biomarkers of Diseases. Pharmacol Ther (2017) 174:63–78. doi: 10.1016/j.pharmthera.2017.02.020 28202367

[29] Boriachek K Islam MN Moller A Salomon C Nam-Trung N Hossain MSA. Biological Functions and Current Advances in Isolation and Detection Strategies for Exosome Nanovesicles. Small (2018) 14(6):1702153. doi: 10.1002/smll.201702153 29282861

[30] Kowal J Tkach M Thery C. Biogenesis and Secretion of Exosomes. Curr Opin Cell Biol (2014) 29:116–25. doi: 10.1016/j.ceb.2014.05.004 24959705

[31] Trajkovic K Hsu C Chiantia S Rajendran L Wenzel D Wieland F. Ceramide Triggers Budding of Exosome Vesicles Into Multivesicular Endosomes. Science (2008) 319(5867):1244–7. doi: 10.1126/science.1153124 18309083

[32] Console L Scalise M Indiveri C. Exosomes in Inflammation and Role as Biomarkers. Clin Chim Acta (2019) 488:165–71. doi: 10.1016/j.cca.2018.11.009 30419221

[33] Li X Corbett AL Taatizadeh E Tasnim N Little JP Garnis C. Challenges and Opportunities in Exosome Research-Perspectives From Biology, Engineering, and Cancer Therapy. Apl Bioeng (2019) 3(1):011503. doi: 10.1063/1.5087122 31069333 PMC6481742

[34] Wolf P. The Nature and Significance of Platelet Products in Human Plasma. Br J Haematol (1967) 13(3):269–88. doi: 10.1111/j.1365-2141.1967.tb08741.x 6025241

[35] van Niel G D'Angelo G Raposo G. Shedding Light on the Cell Biology of Extracellular Vesicles. Nat Rev Mol Cell Biol (2018) 19(4):213–28. doi: 10.1038/nrm.2017.125 29339798

[36] Xu X Lai Y Hua Z-C. Apoptosis and Apoptotic Body: Disease Message and Therapeutic Target Potentials. Biosci Rep (2019) 39:BSR20180992. doi: 10.1042/BSR20180992 30530866 PMC6340950

[37] Samos J Garcia-Olmo DC Picazo MG Rubio-Vitaller A Garcia-Olmo D. Circulating Nucleic Acids in Plasma/Serum and Tumor Progression: Are Apoptotic Bodies Involved? An Experimental Study in a Rat Cancer Model. Ann NY Acad Sci (2006) 1075:165–73. doi: 10.1196/annals.1368.022 17108207

[38] Cao H. Adipocytokines in Obesity and Metabolic Disease. J Endocrinol (2014) 220(2):T47–59. doi: 10.1530/JOE-13-0339 PMC3887367 24403378

[39] Zhou Z Tao Y Zhao H Wang Q. Adipose Extracellular Vesicles: Messengers From and to Macrophages in Regulating Immunometabolic Homeostasis or Disorders. Front Immunol (2021) 12. doi: 10.3389/fimmu.2021.666344 PMC8183682 34108967

[40] Obata Y Kita S Koyama Y Fukuda S Takeda H Takahashi M. Adiponectin/T-Cadherin System Enhances Exosome Biogenesis and Decreases Cellular Ceramides by Exosomal Release. JCI Insight (2018) 3(8):e99680. doi: 10.1172/jci.insight.99680 PMC5931116 29669945

[41] Wei W-Y Zhang N Tang Q-Z. The Potential Role of PPAR Gamma in Obesity-Induced Adipose Tissue Inflammation. Int J Cardiol (2018) 266:220–. doi: 10.1016/j.ijcard.2017.11.016 29887452

[42] AlZaim I Hammoud SH Al-Koussa H Ghazi A Eid AH El-Yazbi AF. Adipose Tissue Immunomodulation: A Novel Therapeutic Approach in Cardiovascular and Metabolic Diseases. Front Cardiovasc Med (2020) 7. doi: 10.3389/fcvm.2020.602088 PMC7705180 33282920

[43] Davis KE Neinast MD Sun K Skiles WM Bills JD Zehr JA. The Sexually Dimorphic Role of Adipose and Adipocyte Estrogen Receptors in Modulating Adipose Tissue Expansion, Inflammation, and Fibrosis. Mol Metab (2013) 2(3):227–42. doi: 10.1016/j.molmet.2013.05.006 PMC3773827 24049737

[44] Goodwill AG. Perivascular Adipose Tissue and Inflammation. Obesity (2016) 24(3):547–. doi: 10.1002/oby.21426 PMC4769115 26854327

[45] Kunz HE Hart CR Gries KJ Parvizi M Laurenti M Dalla Man C. Adipose Tissue Macrophage Populations and Inflammation are Associated With Systemic Inflammation and Insulin Resistance in Obesity. Am J Physiol-Endocrinol Metab (2021) 321(1):E105–E21. doi: 10.1152/ajpendo.00070.2021 PMC8321823 33998291

[46] Connolly KD Guschina IA Yeung V Clayton A Draman MS Von Ruhland C. Characterisation of Adipocyte-Derived Extracellular Vesicles Released Pre- and Post-Adipogenesis. J Extracell Vesicles (2015) 4:29159–. doi: 10.3402/jev.v4.29159 PMC4661001 26609807

[47] Le Jemtel TH Samson R Milligan G Jaiswal A Oparil S. Visceral Adipose Tissue Accumulation and Residual Cardiovascular Risk. Curr Hypertens Rep (2018) 20(9):77. doi: 10.1007/s11906-018-0880-0 29992362

[48] Ying W Riopel M Bandyopadhyay G Dong Y Birmingham A Seo JB. Adipose Tissue Macrophage-Derived Exosomal miRNAs Can Modulate In Vivo and *In Vitro* Insulin Sensitivity. Cell (2017) 171(2):372–84.e12. doi: 10.1016/j.cell.2017.08.035 28942920

[49] Castano C Kalko S Novials A Parrizas M. Obesity-Associated Exosomal miRNAs Modulate Glucose and Lipid Metabolism in Mice. Proc Natl Acad Sci USA (2018) 115(48):12158–63. doi: 10.1073/pnas.1808855115 PMC6275521 30429322

[50] Wen Z Li J Fu Y Zheng Y Ma M Wang C. Hypertrophic Adipocyte-Derived Exosomal miR-802-5p Contributes to Insulin Resistance in Cardiac Myocytes Through Targeting Hsp60. Obesity (2020) 28(10):1932–40. doi: 10.1002/oby.22932 32844579

[51] Mashouri L Yousefi H Aref AR Ahadi AM Molaei F Alahari SK. Exosomes: Composition, Biogenesis, and Mechanisms in Cancer Metastasis and Drug Resistance. Mol Cancer (2019) 18:75. doi: 10.1186/s12943-019-0991-5 30940145 PMC6444571

[52] Kwan HY Chen M Xu K Chen B. The Impact of Obesity on Adipocyte-Derived Extracellular Vesicles. Cell Mol Life Sci (2021) 78(23):7275–88. doi: 10.1007/s00018-021-03973-w PMC8531905 34677643

[53] Yan C Tian X Li J Liu D Ye D Xie Z. A High-Fat Diet Attenuates AMPK Alpha 1 in Adipocytes to Induce Exosome Shedding and Nonalcoholic Fatty Liver Development In Vivo. Diabetes (2021) 70(2):577–88. doi: 10.2337/db20-0146 PMC7881856 33262120

[54] Soley L Falank C Reagan MR. MicroRNA Transfer Between Bone Marrow Adipose and Multiple Myeloma Cells. Curr Osteoporos Rep (2017) 15(3):162–70. doi: 10.1007/s11914-017-0360-5 PMC5479136 28432594

[55] Crewe C Joffin N Rutkowski JM Kim M Zhang F Towler DA. An Endothelial-To-Adipocyte Extracellular Vesicle Axis Governed by Metabolic State. Cell (2018) 175(3):695–708.e13. doi: 10.1016/j.cell.2018.09.005 30293865 PMC6195477

[56] Forghani A Koduru SV Chen C Leberfinger AN Ravnic DJ Hayes DJ. Differentiation of Adipose Tissue-Derived CD34+/CD31- Cells Into Endothelial Cells In Vitro. Regener Eng Trans Med (2020) 6(1):101–10. doi: 10.1007/s40883-019-00093-7 PMC7747864 33344757

[57] Park BW Hah YS Kim JH Cho HY Jung MH Kim DR. Use of Human Adipose Tissue as a Source of Endothelial Cells. Maxillofac Plastic Reconstruct Surg (2010) 32(4):299–305. doi: 10.704/000377.2010.32.4.007

[58] Dai M Yu M Zhang Y Tian W. Exosome-Like Vesicles Derived From Adipose Tissue Provide Biochemical Cues for Adipose Tissue Regeneration. Tissue Eng Part A (2017) 23(21-22):1221–30. doi: 10.1089/ten.tea.2017.0045 28457190

[59] Hutley LJ Herington AC Shurety W Cheung C Vesey DA Cameron DP. Human Adipose Tissue Endothelial Cells Promote Preadipocyte Proliferation. Am J Physiol-Endocrinol Metab (2001) 281(5):E1037–E44. doi: 10.1152/ajpendo.2001.281.5.E1037 11595661

[60] Song M Han L Chen F-F Wang D Wang F Zhang L. Adipocyte-Derived Exosomes Carrying Sonic Hedgehog Mediate M1 Macrophage Polarization-Induced Insulin Resistance *via* Ptch and PI3K Pathways. Cell Physiol Biochem (2018) 48(4):1416–32. doi: 10.1159/000492252 30064125

[61] Lin Y Chen X Tian W Yan Z Zheng X. Cultivation and Morphological Characteristics of Rat Adipose Tissue-Derived Vascular Endothelial Cells In Vitro. Sheng Wu Yi Xue Gong Cheng Xue Za Zhi J Biomed Eng Shengwu Yixue Gongchengxue Zazhi (2006) 23(4):836–8. doi: 10.3321/j.issn:1001-5515.2006.04.034 17002120

[62] Monsuur HN Weijers EM Niessen FB Gefen A Koolwijk P Gibbs S. Extensive Characterization and Comparison of Endothelial Cells Derived From Dermis and Adipose Tissue: Potential Use in Tissue Engineering. PloS One (2016) 11(11):e0167056. doi: 10.1371/journal.pone.0167056 27902740 PMC5130240

[63] Sun Y Shi H Yin S Ji C Zhang X Zhang B. Human Mesenchymal Stem Cell Derived Exosomes Alleviate Type 2 Diabetes Mellitus by Reversing Peripheral Insulin Resistance and Relieving Beta-Cell Destruction. ACS Nano (2018) 12(8):7613–28. doi: 10.1021/acsnano.7b07643 30052036

[64] Wang C Zhang C Liu L Xi A Chen B Li Y. Macrophage-Derived Mir-155-Containing Exosomes Suppress Fibroblast Proliferation and Promote Fibroblast Inflammation During Cardiac Injury. Mol Ther (2017) 25(1):192–204. doi: 10.1016/j.ymthe.2016.09.001 28129114 PMC5363311

[65] Lin X Qin Y Jia J Lin T Lin X Chen L. MiR-155 Enhances Insulin Sensitivity by Coordinated Regulation of Multiple Genes in Mice. PloS Genet (2016) 12(10):e1006308. doi: 10.1371/journal.pgen.1006308 27711113 PMC5053416

[66] Lei Q-Q Huang Y Li B Han L Lv C. MiR-155-5p Promotes Metastasis and Epithelial-Mesenchymal Transition of Renal Cell Carcinoma by Targeting Apoptosis-Inducing Factor. Int J Biol Markers (2021) 36(1):20–7. doi: 10.1177/1724600820978229 33325278

[67] Li H Wang Q Shi J Meng L Chen G. Targeting miR-155 Inhibits Survival of Melanoma Cells by Upregulating FOXO3a. Int J Clin Exp Pathol (2017) 10(3):2988–96. doi: 10.3233/CBM-190555

[68] Howlader M Sultana MI Akter F Hossain MM. Adiponectin Gene Polymorphisms Associated With Diabetes Mellitus: A Descriptive Review. Heliyon (2021) 7(8):e07851. doi: 10.1016/j.heliyon.2021.e07851 34471717 PMC8387910

[69] Kaimala S Kumar CA Allouh MZ Ansari SA Emerald BS. Epigenetic Modifications in Pancreas Development, Diabetes, and Therapeutics. Med Res Rev (2022) 242(3):1343–71. doi: 10.1002/med.21878 PMC9306699 34984701

[70] Mocanu V Timofte D Oboroceanu T Cretu-Silivestru IS Pricope-Veselin A Moraru M. Association of Ghrelin Receptor and Inflammation in Peri-Atrial Adipose Tissue From Obese Patients With Postoperative Atrial Fibrillation. Acta Endocrinol-Bucharest (2020) 16(3):298–302. doi: 10.4183/aeb.2020.298 PMC7748232 33363650

[71] Sierra Rojas JX Garcia-San Frutos M Horrillo D Lauzurica N Oliveros E Maria Carrascosa J. Differential Development of Inflammation and Insulin Resistance in Different Adipose Tissue Depots Along Aging in Wistar Rats: Effects of Caloric Restriction. J Gerontol Ser A-Biol Sci Med Sci (2016) 71(3):310–22. doi: 10.1093/gerona/glv117 26419977

[72] Tam CS Redman LM. Adipose Tissue Inflammation and Metabolic Dysfunction: A Clinical Perspective. Horm Mol Biol Clin Invest (2013) 15(1):19–24. doi: 10.1515/hmbci-2013-0032 PMC6913892 25436729

[73] Wang Q Wang Y Xu D. The Roles of T Cells in Obese Adipose Tissue Inflammation. Adipocyte (2021) 10(1):435–45. doi: 10.1080/21623945.2021.1965314 PMC8463033 34515616

[74] Villarroya F Cereijo R Gavalda-Navarro A Villarroya J Giralt M. Inflammation of Brown/Beige Adipose Tissues in Obesity and Metabolic Disease. J Intern Med (2018) 284(5):492–504. doi: 10.1111/joim.12803 29923291

[75] Chawla A Nguyen KD Goh YPS. Macrophage-Mediated Inflammation in Metabolic Disease. Nat Rev Immunol (2011) 11(11):738–49. doi: 10.1038/nri3071 PMC3383854 21984069

[76] Wei M Gao X Liu L Li Z Wan Z Dong Y. Visceral Adipose Tissue Derived Exosomes Exacerbate Colitis Severity *via* Pro-Inflammatory MiRNAs in High Fat Diet Fed Mice. ACS Nano (2020) 14(4):5099–110. doi: 10.1021/acsnano.0c01860 32275391

[77] Pan Y Hui X Hoo RLC Ye D Chan CYC Feng T. Adipocyte-Secreted Exosomal microRNA-34a Inhibits M2 Macrophage Polarization to Promote Obesity-Induced Adipose Inflammation. J Clin Invest (2019) 129(2):834–49. doi: 10.1172/JCI123069 PMC6355214 30667374

[78] Flier JS. Starvation in the Midst of Plenty: Reflections on the History and Biology of Insulin and Leptin. Endocr Rev (2019) 40(1):1–16. doi: 10.1210/er.2018-00179 30357355 PMC6270967

[79] Ohashi K Ouchi N Matsuzawa Y. Anti-Inflammatory and Anti-Atherogenic Properties of Adiponectin. Biochimie (2012) 94(10):2137–42. doi: 10.1016/j.biochi.2012.06.008 22713764

[80] Moreno-Aliaga MJ Perez-Echarri N Marcos-Gomez B Larequi E Javier Gil-Bea F Viollet B. Cardiotrophin-1 Is a Key Regulator of Glucose and Lipid Metabolism. Cell Metab (2011) 14(2):242–53. doi: 10.1016/j.cmet.2011.05.013 21803294

[81] Tan BK Adya R Farhatullah S Lewandowski KC O'Hare P Lehnert H. Omentin-1, a Novel Adipokine, Is Decreased in Overweight Insulin-Resistant Women With Polycystic Ovary Syndrome - Ex Vivo and *In Vivo* Regulation of Omentin-1 by Insulin and Glucose. Diabetes (2008) 57(4):801–8. doi: 10.2337/db07-0990 18174521

[82] Batista C Yang R-Z Lee M-J Glynn NM Yu D-Z Pray J. Omentin Plasma Levels and Gene Expression Are Decreased in Obesity. Diabetes (2007) 56(6):1655–61. doi: 10.2337/db06-1506 17329619

[83] Yang R-Z Lee M-J Hu H Pray J Wu H-B Hansen BC. Identification of Omentin as a Novel Depot-Specific Adipokine in Human Adipose Tissue: Possible Role in Modulating Insulin Action. Am J Physiol-Endocrinol Metab (2006) 290(6):E1253–E61. doi: 10.1152/ajpendo.00572.2004 16531507

[84] Hernandez-Diaz A Arana-Martinez JC Carbo R Espinosa-Cervantes R Sanchez-Munoz F. Omentin: Role in Insulin Resistance, Inflammation and Cardiovascular Protection. Archiv Cardiol Mexico (2016) 86(3):233–43. doi: 10.1016/j.acmx.2015.09.010 26778502

[85] Cho YK Kang YM Lee SE Lee YL Seol SM Lee WJ. Effect of SFRP5 (Secreted Frizzled-Related Protein 5) on the WNT5A (Wingless-Type Family Member 5a)-Induced Endothelial Dysfunction and Its Relevance With Arterial Stiffness in Human Subjects. Arterioscler Thromb Vasc Biol (2018) 38(6):1358–67. doi: 10.1161/ATVBAHA.117.310649 29674475

[86] Carstensen-Kirberg M Kannenberg JM Huth C Meisinger C Koenig W Heier M. Inverse Associations Between Serum Levels of Secreted Frizzled-Related Protein-5 (SFRP5) and Multiple Cardiometabolic Risk Factors: KORA F4 Study. Cardiovasc Diabetol (2017) 16:109. doi: 10.1186/s12933-017-0591-x 28851362 PMC5574239

[87] Miyoshi T Doi M Usui S Iwamoto M Kajiya M Takeda K. Low Serum Level of Secreted Frizzled-Related Protein 5, an Anti-Inflammatory Adipokine, is Associated With Coronary Artery Disease. Atherosclerosis (2014) 233(2):454–9. doi: 10.1016/j.atherosclerosis.2014.01.019 24530778

[88] Hu Z Deng H Qu H. Plasma SFRP5 Levels are Decreased in Chinese Subjects With Obesity and Type 2 Diabetes and Negatively Correlated With Parameters of Insulin Resistance. Diabetes Res Clin Pract (2013) 99(3):391–5. doi: 10.1016/j.diabres.2012.11.026 23290274

[89] Bartel DP. MicroRNAs: Genomics, Biogenesis, Mechanism, and Function. Cell (2004) 116(2):281–97. doi: 10.1016/S0092-8674(04)00045-5 14744438

[90] Lanzillotti C De Mattei M Mazziotta C Taraballi F Rotondo JC Tognon M. Long Non-Coding RNAs and MicroRNAs Interplay in Osteogenic Differentiation of Mesenchymal Stem Cells. Front Cell Dev Biol (2021) 9. doi: 10.3389/fcell.2021.646032 PMC8063120 33898434

[91] Makarova J Turchinovich A Shkurnikov M Tonevitsky A. Extracellular miRNAs and Cell-Cell Communication: Problems and Prospects. Trends Biochem Sci (2021) 46(8):640–51. doi: 10.1016/j.tibs.2021.01.007 33610425

[92] Prattichizzo F Matacchione G Giuliani A Sabbatinelli J Olivieri F de Candia P. Extracellular Vesicle-Shuttled miRNAs: A Critical Appraisal of Their Potential as Nano-Diagnostics and Nano-Therapeutics in Type 2 Diabetes Mellitus and its Cardiovascular Complications. Theranostics (2021) 11(3):1031–45. doi: 10.7150/thno.51605 PMC7738884 33391519

[93] Wang X He Y Mackowiak B Gao B. MicroRNAs as Regulators, Biomarkers and Therapeutic Targets in Liver Diseases. Gut (2021) 70(4):784–95. doi: 10.1136/gutjnl-2020-322526 33127832

[94] Arroyo JD Chevillet JR Kroh EM Ruf IK Pritchard CC Gibson DF. Argonaute2 Complexes Carry a Population of Circulating microRNAs Independent of Vesicles in Human Plasma. Proc Natl Acad Sci USA (2011) 108(12):5003–8. doi: 10.1073/pnas.1019055108 PMC3064324 21383194

[95] Guduric-Fuchs J O'Connor A Camp B O'Neill CL Medina RJ Simpson DA. Selective Extracellular Vesicle-Mediated Export of an Overlapping Set of microRNAs From Multiple Cell Types. BMC Genomics (2012) 13:357. doi: 10.1186/1471-2164-13-357 22849433 PMC3532190

[96] Kosaka N Iguchi H Hagiwara K Yoshioka Y Takeshita F Ochiya T. Neutral Sphingomyelinase 2 (Nsmase2)-Dependent Exosomal Transfer of Angiogenic MicroRNAs Regulate Cancer Cell Metastasis. J Biol Chem (2013) 288(15):10849–59. doi: 10.1074/jbc.M112.446831 PMC3624465 23439645

[97] Villarroya-Beltri C Gutierrez-Vazquez C Sanchez-Cabo F Perez-Hernandez D Vazquez J Martin-Cofreces N. Sumoylated Hnrnpa2b1 Controls the Sorting of miRNAs Into Exosomes Through Binding to Specific Motifs. Nat Commun (2013) 4:2980. doi: 10.1038/ncomms3980 24356509 PMC3905700

[98] Pan Z Zhao R Li B Qi Y Qiu W Guo Q. EWSR1-Induced Circneil3 Promotes Glioma Progression and Exosome-Mediated Macrophage Immunosuppressive Polarization via Stabilizing IGF2BP3. Mol Cancer (2022) 21(1):16. doi: 10.1186/s12943-021-01485-6 35031058 PMC8759291

[99] Koppers-Lalic D Hackenberg M Bijnsdorp IV van Eijndhoven MAJ Sadek P Sie D. Nontemplated Nucleotide Additions Distinguish the Small RNA Composition in Cells From Exosomes. Cell Rep (2014) 8(6):1649–58. doi: 10.1016/j.celrep.2014.08.027 25242326

[100] Zhang J Li S Li L Li M Guo C Yao J. Exosome and Exosomal MicroRNA: Trafficking, Sorting, and Function. Genomics Proteomics Bioinf (2015) 13(1):17–24. doi: 10.1016/j.gpb.2015.02.001 PMC4411500 25724326

[101] Liu X-M Ma L Schekman R. Selective Sorting of microRNAs Into Exosomes by Phase-Separated YBX1 Condensates. Elife (2021) 10:e71982. doi: 10.7554/eLife.71982 34766549 PMC8612733

[102] Garcia-Martin R Wang G Brandao BB Zanotto TM Shah S Patel SK. MicroRNA Sequence Codes for Small Extracellular Vesicle Release and Cellular Retention. Nature (2022) 601(7893):446–51. doi: 10.1038/s41586-021-04234-3 PMC9035265 34937935

[103] Zhang S Cheng Z Wang Y Han T. The Risks of miRNA Therapeutics: In a Drug Target Perspective. Drug Design Dev Ther (2021) 15:721–33. doi: 10.2147/DDDT.S288859 PMC7910153 33654378

[104] Thomou T Mori MA Dreyfuss JM Konishi M Sakaguchi M Wolfrum C. Adipose-Derived Circulating miRNAs Regulate Gene Expression in Other Tissues. Nature (2017) 542(7642):450–5. doi: 10.1038/nature21365 PMC5330251 28199304

[105] Garcia-Martin R Brandao BB Thomou T Altindis E Kahn CR. Tissue Differences in the Exosomal/Small Extracellular Vesicle Proteome and Their Potential as Indicators of Altered Tissue Metabolism. Cell Rep (2022) 38(3):110277. doi: 10.1016/j.celrep.2021.110277 PMC8867597 35045290

[106] Zhang Y Yu M Dai M Chen C Tang Q Jing W. miR-450a-5p Within Rat Adipose Tissue Exosome-Like Vesicles Promotes Adipogenic Differentiation by Targeting WISP2. J Cell Sci (2017) 130(6):1158–68. doi: 10.1242/jcs.197764 28167681

[107] Jansen F Yang X Hoelscher M Cattelan A Schmitz T Proebsting S. Endothelial Microparticle-Mediated Transfer of MicroRNA-126 Promotes Vascular Endothelial Cell Repair *via* SPRED1 and Is Abrogated in Glucose-Damaged Endothelial Microparticles. Circulation (2013) 128(18):2026–38. doi: 10.1161/CIRCULATIONAHA.113.001720 24014835

[108] Jansen F Wang H Przybilla D Franklin BS Dolf A Pfeifer P. Vascular Endothelial Microparticles-Incorporated microRNAs are Altered in Patients With Diabetes Mellitus. Cardiovasc Diabetol (2016) 15:49. doi: 10.1186/s12933-016-0367-8 27005938 PMC4804519

[109] Chen M-T Zhao Y-T Zhou L-Y Li M Zhang Q Han Q. Exosomes Derived From Human Umbilical Cord Mesenchymal Stem Cells Enhance Insulin Sensitivity in Insulin Resistant Human Adipocytes. Curr Med Sci (2021) 41(1):87–93. doi: 10.1007/s11596-021-2323-4 33582911

[110] Feng X Wang Z Fillmore R Xi Y. MiR-200, a New Star miRNA in Human Cancer. Cancer Lett (2014) 344(2):166–73. doi: 10.1016/j.canlet.2013.11.004 PMC3946634 24262661

[111] Horiguchi M Okada Y Turudome Y Ushijima K. Exosome Degeneration in Mesenchymal Stem Cells Derived From Patients With Type 1 Diabetes Mellitus. Int J Mol Sci (2021) 22(20):10906. doi: 10.3390/ijms222010906 34681566 PMC8536020

[112] Liu Z Gan L Zhang T Ren Q Sun C. Melatonin Alleviates Adipose Inflammation Through Elevating -Ketoglutarate and Diverting Adipose-Derived Exosomes to Macrophages in Mice. J Pineal Res (2018) 64(1):e12455. doi: 10.1111/jpi.12455 29149454

[113] Zhang X Jiang Y Huang Q Wu Z Pu H Xu Z. Exosomes Derived From Adipose-Derived Stem Cells Overexpressing Glyoxalase-1 Protect Endothelial Cells and Enhance Angiogenesis in Type 2 Diabetic Mice With Limb Ischemia. Stem Cell Res Ther (2021) 12(1):403. doi: 10.1186/s13287-021-02475-7 34266474 PMC8281719

[114] Volz A-C Huber B Kluger PJ. Adipose-Derived Stem Cell Differentiation as a Basic Tool for Vascularized Adipose Tissue Engineering. Differentiation (2016) 92(1-2):52–64. doi: 10.1016/j.diff.2016.02.003 26976717

[115] Patel RS Carter G El Bassit G Patel AA Cooper DR Murr M. Adipose-Derived Stem Cells From Lean and Obese Humans Show Depot Specific Differences in Their Stem Cell Markers, Exosome Contents and Senescence: Role of Protein Kinase C Delta (PKCdelta) in Adipose Stem Cell Niche. Stem Cell Invest (2016) 3:2. doi: 10.3978/j.issn.2306-9759.2016.01.02 PMC4923648 27358894

[116] Zhu LL Huang X Yu W Chen H Chen Y Dai YT. Transplantation of Adipose Tissue-Derived Stem Cell-Derived Exosomes Ameliorates Erectile Function in Diabetic Rats. Andrologia (2018) 50(2):e12871. doi: 10.1111/and.12871 29057541

[117] Wang S Olson EN. AngiomiRs-Key Regulators of Angiogenesis. Curr Opin Genet Dev (2009) 19(3):205–11. doi: 10.1016/j.gde.2009.04.002 PMC2696563 19446450

[118] Shen T Zheng QQ Shen J Li QS Song XH Luo HB. Effects of Adipose-Derived Mesenchymal Stem Cell Exosomes on Corneal Stromal Fibroblast Viability and Extracellular Matrix Synthesis (Vol 131, Pg 704, 2018). Chin Med J (2021) 134(11):1375. doi: 10.1097/CM9.0000000000001481 PMC5865317 29521294

[119] Zhao H Shang Q Pan Z Bai Y Li Z Zhang H. Exosomes From Adipose-Derived Stem Cells Attenuate Adipose Inflammation and Obesity Through Polarizing M2 Macrophages and Beiging in White Adipose Tissue. Diabetes (2018) 67(2):235–47. doi: 10.2337/db17-0356 29133512

[120] Shang Q Bai Y Wang G Song Q Guo C Zhang L. Delivery of Adipose-Derived Stem Cells Attenuates Adipose Tissue Inflammation and Insulin Resistance in Obese Mice Through Remodeling Macrophage Phenotypes. Stem Cells Dev (2015) 24(17):2052–64. doi: 10.1089/scd.2014.0557 25923535

[121] Al-Ghadban S Bunnell BA. Adipose Tissue-Derived Stem Cells: Immunomodulatory Effects and Therapeutic Potential. Physiology (2020) 35(2):125–33. doi: 10.1152/physiol.00021.2019 32027561

[122] Alonso-Alonso ML Garcia-Posadas L Diebold Y. Extracellular Vesicles From Human Adipose-Derived Mesenchymal Stem Cells: A Review of Common Cargos. Stem Cell Rev Rep (2021) 854–901. doi: 10.1007/s12015-021-10155-5 33904115 PMC8942954

[123] Hong P Yang H Wu Y Li K Tang Z. The Functions and Clinical Application Potential of Exosomes Derived From Adipose Mesenchymal Stem Cells: A Comprehensive Review. Stem Cell Res Ther (2019) 10(1):242. doi: 10.1186/s13287-019-1358-y 31391108 PMC6686455

[124] Li X Xie X Lian W Shi R Han S Zhang H. Exosomes From Adipose-Derived Stem Cells Overexpressing Nrf2 Accelerate Cutaneous Wound Healing by Promoting Vascularization in a Diabetic Foot Ulcer Rat Model. Exp Mol Med (2018) 50(4):1–14. doi: 10.1038/s12276-018-0058-5 PMC5938041 29651102

[125] Shafei S Khanmohammadi M Heidari R Ghanbari H Nooshabadi VT Farzamfar S. Exosome Loaded Alginate Hydrogel Promotes Tissue Regeneration in Full-Thickness Skin Wounds: An *In Vivo* Study. J Biomed Mater Res Part A (2020) 108(3):545–56. doi: 10.1002/jbm.a.36835 31702867

[126] Bosch S de Beaurepaire L Allard M Mosser M Heichette C Chretien D. Trehalose Prevents Aggregation of Exosomes and Cryodamage. Sci Rep (2016) 6:36162. doi: 10.1038/srep36162 27824088 PMC5099918

[127] Gonzalez-Cubero E Gonzalez-Fernandez ML Gutierrez-Velasco L Navarro-Ramirez E Villar-Suarez V. Isolation and Characterization of Exosomes From Adipose Tissue-Derived Mesenchymal Stem Cells. J Anatomy (2021) 238(5):1203–17. doi: 10.1111/joa.13365 PMC8053584 33372709

[128] Patel GK Khan MA Zubair H Srivastava SK Khushman M Singh S. Comparative Analysis of Exosome Isolation Methods Using Culture Supernatant for Optimum Yield, Purity and Downstream Applications. Sci Rep (2019) 9(1):5335. doi: 10.1038/s41598-019-41800-2 30926864 PMC6441044

[129] Zhang Y Bi J Huang J Tang Y Du S Li P. Exosome: A Review of Its Classification, Isolation Techniques, Storage, Diagnostic and Targeted Therapy Applications. Int J Nanomed (2020) 15:6917–34. doi: 10.2147/IJN.S264498 PMC7519827 33061359

[130] O'Brien K Breyne K Ughetto S Laurent LC Breakefield XO. RNA Delivery by Extracellular Vesicles in Mammalian Cells and its Applications. Nat Rev Mol Cell Biol (2020) 21(10):585–606. doi: 10.1038/s41580-020-0251-y 32457507 PMC7249041

